# A novel method for producing target cells and assessing cytotoxic T lymphocyte activity in outbred hosts

**DOI:** 10.1186/1472-6750-9-18

**Published:** 2009-03-11

**Authors:** Francesca Bonci, Elisa Zabogli, Francesca Conti, Antonio Merico, Giulia Freer, Mauro Bendinelli, Mauro Pistello

**Affiliations:** 1 Retrovirus Center and Virology Section, Department of Experimental Pathology, University of Pisa, Via San Zeno, 35, 56127 Pisa, Italy

## Abstract

**Background:**

Cytotoxic T lymphocytes play a crucial role in the immunological control of microbial infections and in the design of vaccines and immunotherapies. Measurement of cytotoxic T lymphocyte activity requires that the test antigen is presented by target cells having the same or compatible class I major hystocompatibility complex antigens as the effector cells. Conventional assays use target cells labeled with ^51^chromium and infer cytotoxic T lymphocyte activity by measuring the isotope released by the target cells lysed following incubation with antigen-specific cytotoxic T lymphocytes. This assay is sensitive but needs manipulation and disposal of hazardous radioactive reagents and provides a bulk estimate of the reporter released, which may be influenced by spontaneous release of the label and other poorly controllable variables. Here we describe a novel method for producing target in outbred hosts and assessing cytotoxic T lymphocyte activity by flow cytometry.

**Results:**

The method consists of culturing skin fibroblasts, immortalizing them with a replication defective clone of simian virus 40, and finally transducing them with a bicistronic vector encoding the target antigen and the reporter green fluorescent protein. When used in a flow cytometry-based assay, the target cells obtained with this method proved valuable for assessing the viral envelope protein specific cytotoxic T lymphocyte activity in domestic cats acutely or chronically infected with feline immunodeficiency virus, a lentivirus similar to human immunodeficiency virus and used as animal model for AIDS studies.

**Conclusion:**

Given the versatility of the bicistronic vector used, its ability to deliver multiple and large transgenes in target cells, and its extremely wide cell specificity when pseudotyped with the vesicular stomatitis virus envelope protein, the method is potentially exploitable in many animal species.

## Background

Cytotoxic T lymphocytes (CTLs) are key components of the cell-mediated immune responses and play an essential role in protection from and containment of a variety of pathogens [1]. Since CTLs have been shown to play a major role in the control of human immunodeficiency virus (HIV) and other lentiviruses [2,3], measurement of their activity has become an important parameter for testing the efficacy of candidate vaccines and other immunologic interventions directed against these important agents of disease [4].

CTL assays require that the test antigen is presented by target cells having the same or compatible class I major hystocompatibility complex antigens as the effector cells. This permits to the antigen-specific CTLs to interact with the target cells and determine their lysis, which is then quantitated as a measure of CTL activity. The most used format is the ^51^chromium (^51^Cr)-release assay in which this radioisotope, absorbed by the target cells prior to mixing with the effector cells, is released in the supernatant medium and measured at the end of the incubation period [5]. This assay is sensitive but has the disadvantage of requiring the manipulation and disposal of hazardous radioactive reagents. Thus, over the years, ^51^Cr has been tentatively replaced by various cell dyes with somewhat inconsistent results. However, all these assays have the limitation of providing a bulk estimate of the reporter released, which may be influenced by several, poorly controllable variables, such as inefficient labeling of target cells or spontaneous release of the label [6]. More recently, new flow cytometry-based (F-CTL) assays, using target cells labeled with fluorescent dyes or substrates for caspases or other apoptotic enzymes, have been proposed [7-12]. These assays appear to be as or more sensitive than the standard ^51^Cr methods and, in addition, may permit to precisely count the target cells that have actually been lysed and to identify the phenotype of the effector cells involved [6,9].

Here, we describe a novel method for producing target cells that has provided satisfactory results in an F-CTL assay we have set up to assess envelope (Env)-specific CTL activity in domestic specific pathogen-free (SPF) cats infected with feline immunodeficiency virus (FIV), the feline equivalent of HIV. Since no inbred cats are available, testing CTL activity in this species is dependent on the use of autologous target cells, a factor that has hitherto limited routine measurements of CTL activity [13,14]. The method relies on a bicistronic vector derived from FIV but having very little left of the original virus which is used to transduce immortalized feline skin fibroblasts. Since the vector can be pseudotyped with the different Envs and may have broad tropism [15], the method is potentially exploitable in other animal species as well.

## Results

### Production and characterization of the target cells

**Figure 1:**
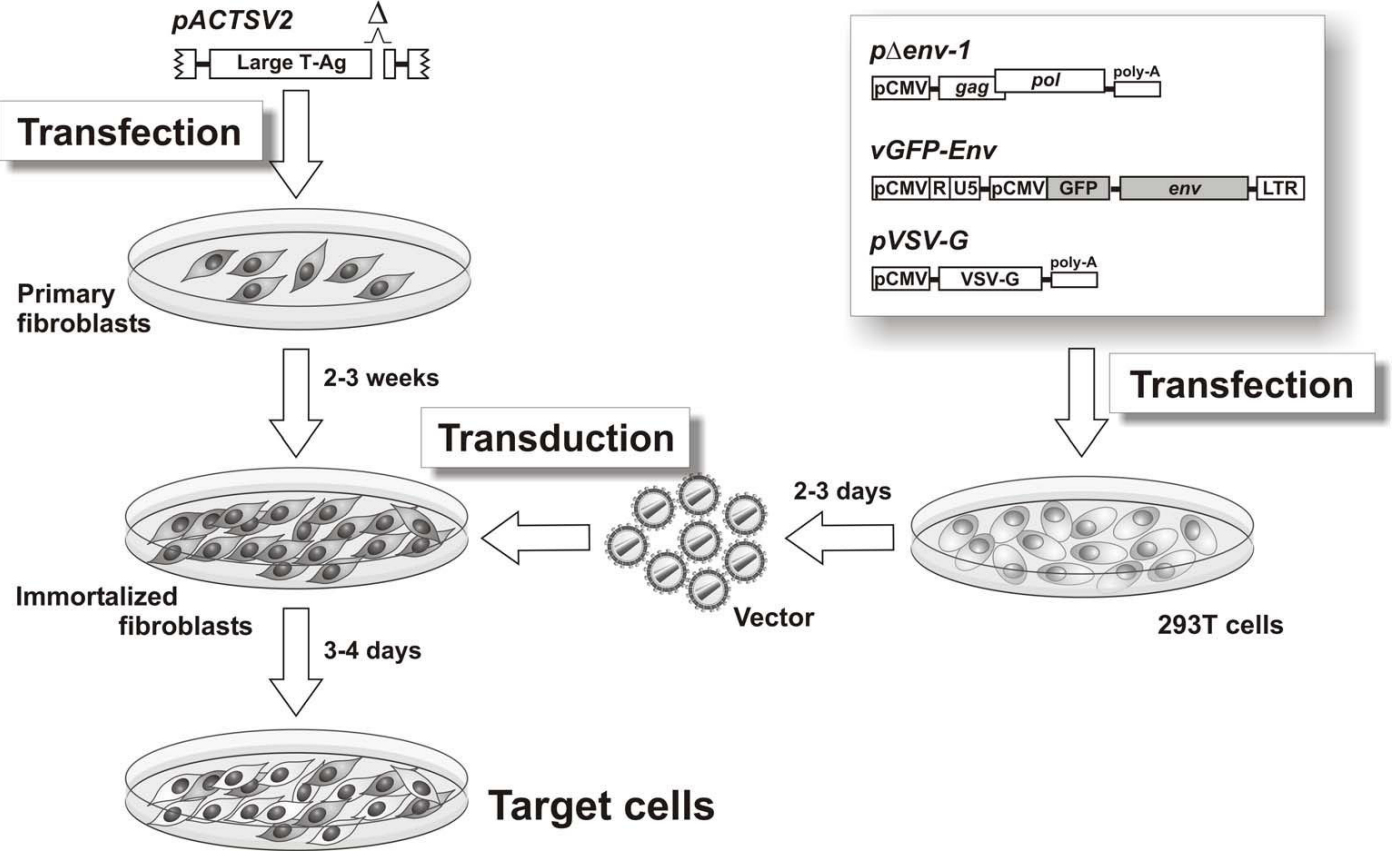
**Outline of target cell production**. Primary feline fibroblasts were immortalized by transfection with plasmid pACTSV2, an SV40 genome encoding a functional small T antigen and an internally deleted large T antigen. The cells thus immortalized were then transduced with vesicular stomatitis virus glycoprotein G (VSV-G) pseudotyped particles produced in 293T cells and delivering target *env *gene and reporter GFP gene.

Production of the target cells expressing the Env of FIV-Petaluma (FIV-Pet) and reporter green fluorescent protein (GFP) is schematically depicted by Figure 1. Primary fibroblasts were obtained by culturing skin biopsies of the study cats and, when confluent, transferred to larger flasks. One flask was then used to control for cell competence for transfection by using a plasmid encoding GFP (pcDNA3-GFP). As expected for primary cells and in agreement with Köksoy et al. [16], the fibroblasts proved moderately transfectable, since by day 2 post transfection GFP expressing cells ranged between 15% and 30% (data not shown). The remaining flasks were transfected with a plasmid encoding a replication defective simian virus 40 (SV40) genome (pACTSV2) and subcultured every 3–4 days for further 3 weeks. Ten to 15 days later, the pACTSV2 transfected cells showed unequivocal signs of immortalization that were maintained for at least 30 passages regardless of whether the cells were kept in culture continuously or repeatedly freeze-stored and cultured again. This contrasted strongly with sister cultures that were left untreated or transfected with pcDNA3-GFP, which reached senescence after 6–8 passages and barely resisted freezing (data not shown). Of note, fibroblasts generated from FIV-infected animals – regardless of whether immortalized or not – showed no evidence of FIV replication, as determined by weekly testing the supernatants for p25 capsid antigen, suggesting that they had been spared from infection or had stopped supporting detectable FIV replication as a result of culture (data not shown).

**Figure 2:**
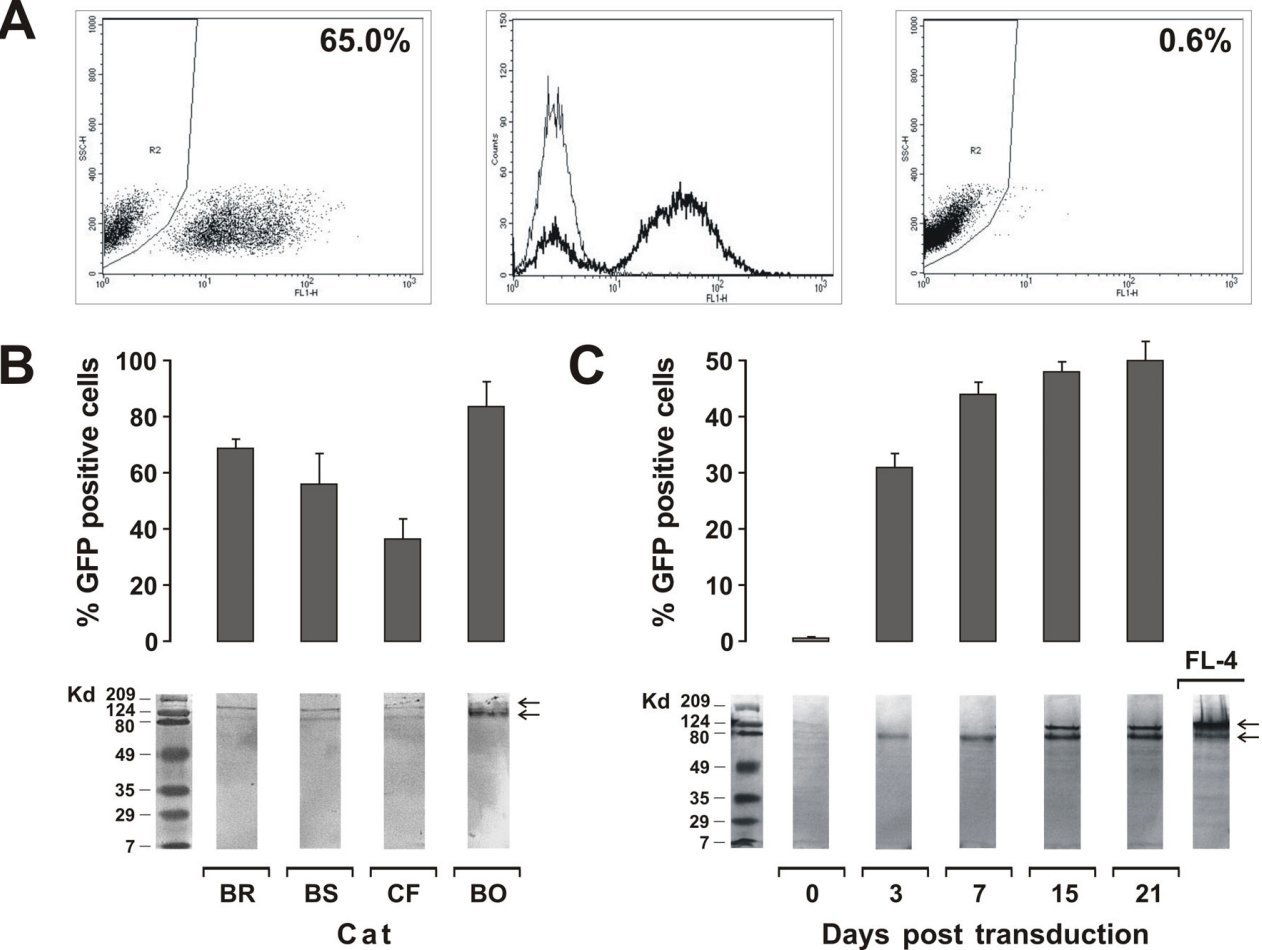
**Efficiency and duration of transduction of immortalized feline fibroblasts by vGFP and vEnv-GFP**. A: GFP expression by the fibroblasts of uninfected cat BO 2 days post-transduction with vGFP (50 TU/cell), as determined by flow cytometry. Left panel, percent GFP positive cells; middle panel, intensity of GFP expression; right panel, mock-transduced cells. B: Susceptibility of fibroblasts from 3 FIV chronically infected cats and 1 uninfected cat to transduction by vEnv-GFP at 100 TU/cell. Percent GFP positive cells (upper panel) and extent of Env expression (lower panel) were evaluated 2 days post-transduction by flow cytometry and western blot, respectively. C: GFP expression (upper panel) and Env expression (lower panel) by fibroblasts of one infected cat (BS) transduced with vEnv-GFP at 50 TU/cell, evaluated at the indicated times by flow cytometry and western blot, respectively. The arrows in panels B and C indicate the Env precursor (135 Kd) and surface (95 Kd) glycoproteins. Bars represent SD calculated from 3 independent experiments. FL-4: Env expressed by the chronically FIV infected cell line FL-4.

Transduction of pACTSV2 immortalized fibroblasts was carried out with viral particles produced in 293T cells and encapsidating the monocystronic vector carrying GFP alone (vGFP) or the bicistronic version delivering Env and GFP (vEnv-GFP) (Figure 1). For a preliminary assessment of the efficiency of transduction, the immortalized fibroblasts of an uninfected cat were exposed to serial 2-fold dilutions of a vGFP preparation and, 2 days later, analyzed for GFP expression by flow cytometry. The proportion of cells transduced proved clearly dose-dependent ranging from 10% with 12.5 transduction units (TU)/cell to over 90% with 200 TU/cell. Importantly, transduced cells fluoresced at high levels (Figure 2A) even after several weeks in culture and when repeatedly stored frozen and recultured (data not shown), indicative of a stable transduction. Different batches of the vectors and fresh or thawed cells yielded similar results. Interestingly, for reasons that were not specifically addressed, immortalized fibroblasts proved consistently more prone to transduction (by a factor of approximately 5) than the non-immortalized cells from which they derived (data not shown).

Unless differently stated, in the subsequent studies the immortalized fibroblasts expressing Env and GFP (Env^+^GFP^+ ^fibroblasts) used as targets were obtained by transducing with 100 TU vEnv-GFP per cell. Figure 2B shows the results obtained with this TU dose in one experiment in which we compared immortalized fibroblasts obtained from 4 cats, one uninfected and 3 chronically FIV infected, for susceptibility to transduction. Although at day 2 post-transduction the percentage of control cells expressing GFP alone (GFP^+ ^fibroblasts) varied somewhat, transduction was successful with the cells of all cats. In addition, both the proportion of transduced cells and the extent they expressed Env had a clear tendency to increase with time, at least for the 3 weeks investigated (Figure 2C). Importantly, the Env expressed by Env^+^GFP^+ ^fibroblasts showed a precursor to surface glycoprotein ratio similar to that of cells chronically infected with FIV, indicating that it was processed correctly (Figure 2C).

### The Env^+^GFP^+ ^fibroblasts as a stimulus for expanding Env-specific CTL activity *ex vivo*

**Figure 3:**
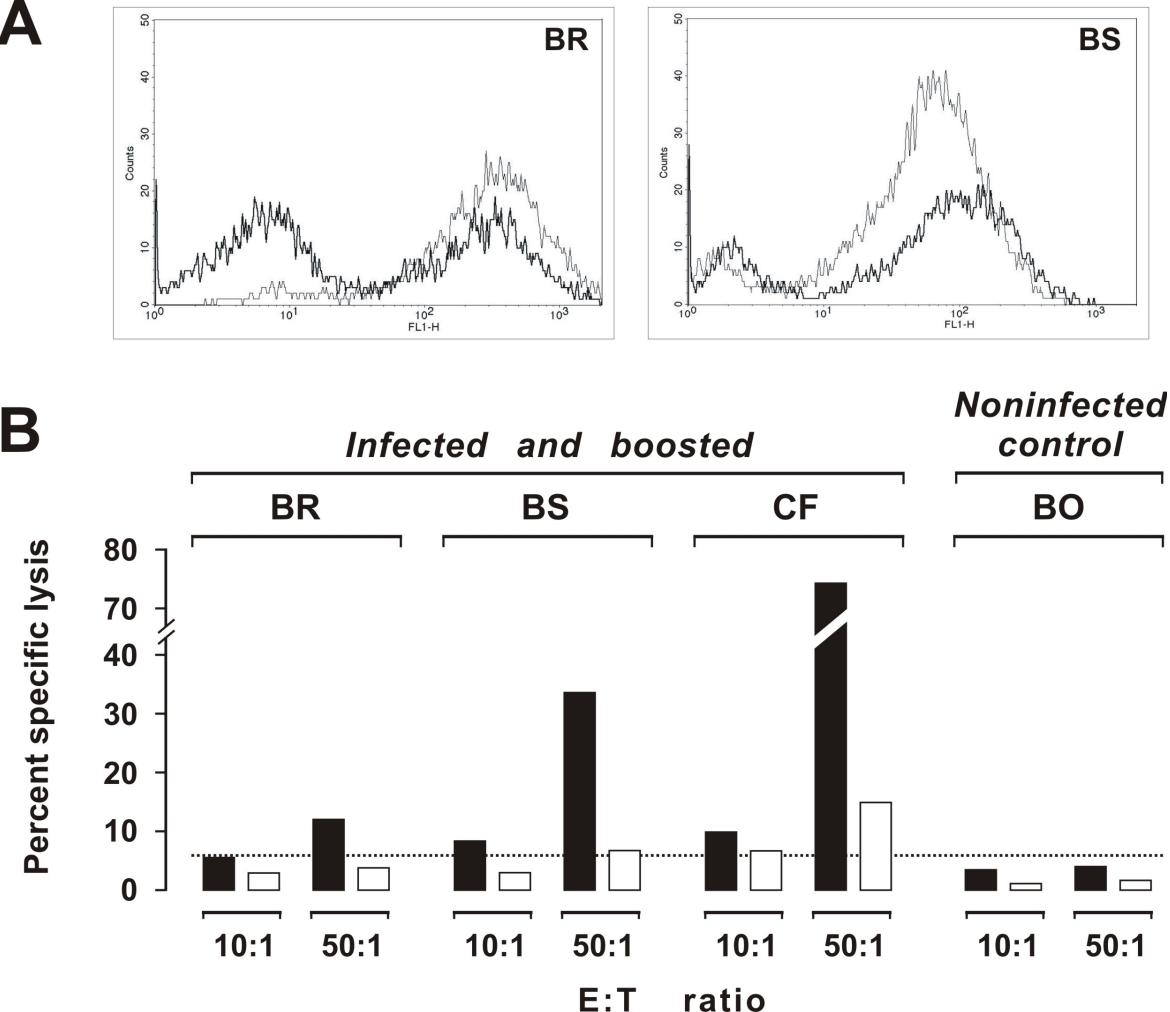
**Flow cytometry plot of the CTL assay and comparative efficiency at restimulating CTL activity *in vitro *of Env transduced and Env peptides loaded immortalized fibroblasts**. A: CTL activity following lymphocyte restimulation with Env-transduced immortalized fibroblasts as measured by F-CTL. Right and left panels show the results obtained with cats BR and BS, respectively. The gray line indicates the target population incubated alone, the thick line indicates residual target cells after incubation with restimulated lymphocytes. B: Comparative efficiency of Env transduced and Env peptides-loaded immortalized fibroblasts at restimulating CTL activity. Prior to the assay, the effector cells (PBMCs of chronically FIV infected cats boosted with p-Env-GMCSF 18 weeks earlier) were restimulated with Env^+^GFP^+ ^fibroblasts (solid columns) or immortalized fibroblasts pulsed for 1 hour with pooled peptides covering the entire Env (empty columns).

In this experiment we investigated if and how effectively the Env^+^GFP^+ ^fibroblasts could be used as a stimulus to expand Env-specific CTL activity *ex vivo *by comparing them with immortalized fibroblasts loaded with a pool of peptides covering the whole FIV Env. This was done by using the peripheral blood mononuclear cells (PBMCs) of BR, BS and CF, 3 chronically FIV infected cats known to possess substantial Env-specific CTL activity as a result of boosting with a DNA immunogen (see below). These PBMCs were co-cultured for 5 days with autologous immortalized fibroblasts that had been transduced with vEnv-GFP or had been pulsed for 1 hour with the pooled peptides or, as controls, with autologous GFP^+ ^fibroblasts or fibroblasts that had not been further manipulated. The PMBCs thus incubated were then tested for CTL activity against Env^+^GFP^+ ^fibroblasts. Lysis by PBMCs restimulated with autologous GFP^+ ^fibroblasts or immortalized fibroblasts was at background levels (data not shown). The Env^+^GFP^+ ^fibroblasts caused an expansion of CTL activity readily measurable (Figure 3A) and substantially higher than the peptide-loaded counterparts (Figure 3B). As expected, either stimulus had no effect on the PBMCs of an uninfected cat used as control.

### The Env^+^GFP^+ ^fibroblasts as targets for measuring Env-specific CTL activity in acutely FIV infected cats

**Figure 4:**
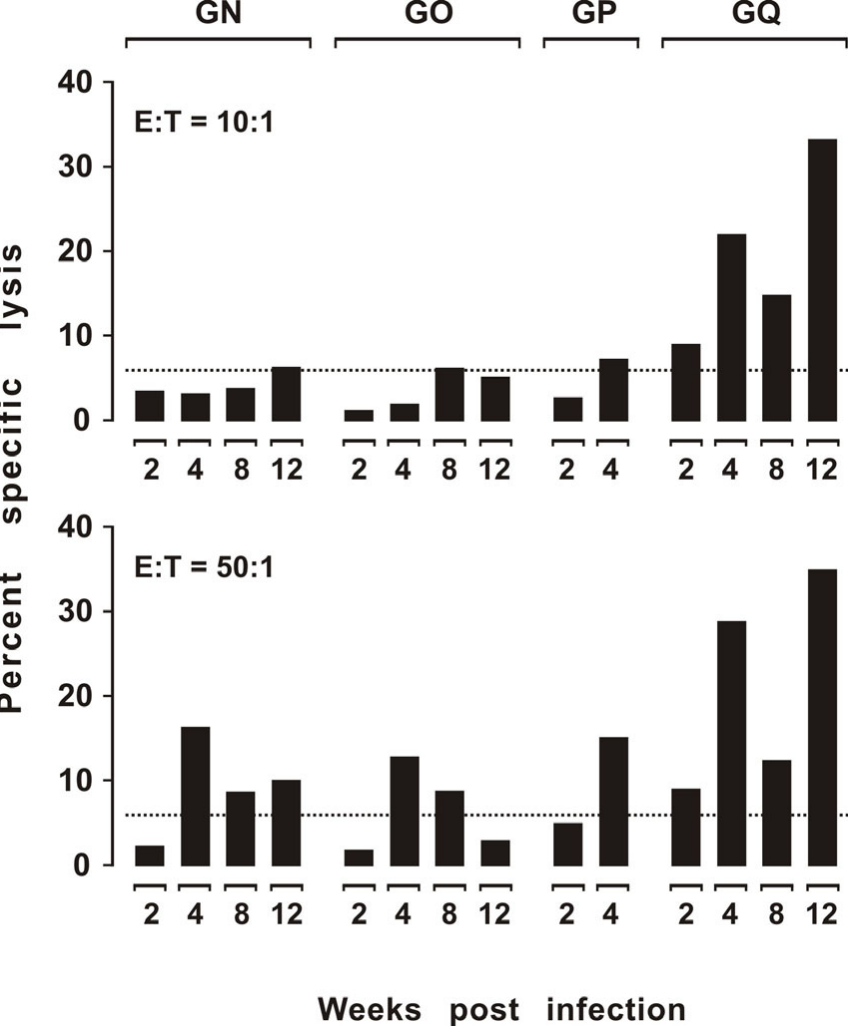
**Env-specific CTL activity in acutely FIV infected cats**. The PBMCs were obtained at the indicated times post-infection and used as effector cells without *ex vivo *restimulation. The dotted line indicates the cut-off value. Cat GP died 40 days post inoculation due to unexplained renal failure.

Collectively, the above data had demonstrated that the Env^+^GFP^+ ^fibroblasts expressed the target and reporter genes in stable and correct fashion. To test whether they were also suitable targets for CTL assays, we employed them in the F-CTL assay using, as effector cells, PBMCs collected from cats during acute FIV infection. Fibroblasts were harvested from SPF cats GN, GO, GP and GQ, processed as above, and frozen. The animals were then inoculated with a dose of FIV-Pet which produced the expected levels of viremia as determined starting 2 weeks post-infection. CTL activity was examined 2, 4, 8 and 12 weeks after infection. The test was carried out both with fresh PBMCs and with PBMCs that, prior to the F-CTL assay, were restimulated *ex vivo *with autologous Env^+^GFP^+ ^fibroblasts as described above. Target cells were autologous Env^+^GFP^+ ^fibroblasts and, as controls, autologous GFP^+ ^fibroblasts and heterologous Env^+^GFP^+ ^fibroblasts; however, significant cell lysis was observed only with the former cells. As depicted by Figure 4, which shows the percent specific lysis values observed with the unstimulated PBMCs at effector:target (E:T) ratios of 1:10 and 1:50, in one cat CTL activity could already be detected 2 weeks post-infection. In the other animals, the test was first found positive at 4 weeks post-infection, usually at the E:T ratio 50:1 only. At later times of infection, this picture did not change significantly, except for the fact that E:T ratio 10:1 yielded a measurable level of lysis more frequently than at earlier times. Percent lysis obtained with *ex vivo *restimulated PBMCs were only marginally higher than obtained with fresh PBMCs (data not shown), suggesting that in acutely infected cats Env-specific CTL activity cannot be significantly expanded *ex vivo*. Of note, cat GQ who exhibited the most prompt and robust CTL response developed levels of plasma viremia, proviral DNA in the PBMC, and a reduction of circulating CD4^+ ^T-lymphocyte counts similar to the other animals (data not shown).

### The Env^+^GFP^+ ^fibroblasts as targets for measuring Env-specific CTL activity in chronically FIV infected cats

**Figure 5:**
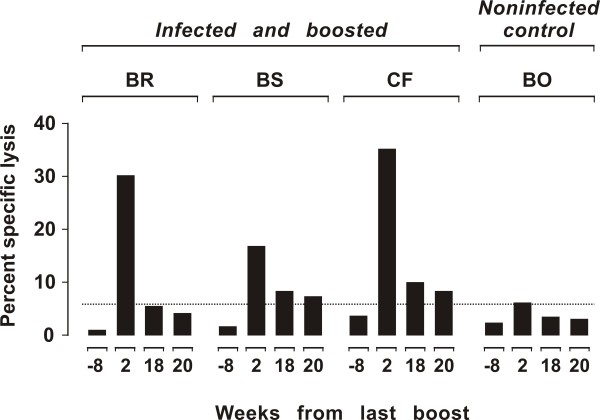
**Env-specific CTL activity in chronically FIV infected cats before and after boosting with a DNA immunogen**. The effector PBMCs were obtained at the indicated times and used at E:T ratio 10:1 after *ex vivo *restimulation with Env^+^GFP^+ ^fibroblasts. The dotted line indicates the cut-off value.

In these experiments, Env^+^GFP^+ ^fibroblasts and F-CTL assay were used to measure anti-Env CTL in the PBMCs of three SPF cats infected with FIV-Pet 5 years earlier. As determined by periodical monitoring, these animals were in a steady state phase of infection since they had stable or slightly fluctuating moderate levels of viremia, high FIV antibodies titers, and moderately reduced circulating CD4^+ ^T-lymphocytes counts (data not shown). A fourth cat (BO), who had been inoculated exactly as the others but had remained consistently FIV negative, was used as noninfected control. Effector PBMCs were either used fresh or restimulated *ex vivo *by co-cultivation with autologous Env^+^GFP^+ ^fibroblasts; however, in no case they exhibited measurable CTL activity (time -8 weeks in Figure 5). An attempt was therefore done to boost anti-Env immune responses of the cats by DNA immunization. Starting 2 weeks after the above CTL assay, the 4 animals were given 3 doses of a plasmid encoding the Env of FIV-Pet and feline granulocyte-macrophage-colony stimulating factor (p-Env-GMCSF), 3 weeks apart, and after 2 further weeks again tested for CTL activity. As shown by Figure 5, the uninfected control cat showed no evidence that this immunization had elicited significant CTL activity. In contrast, all 3 FIV chronically infected animals exhibited robust CTL activity, ranging between 16% and 35% at E:T ratio 10:1. When the test was repeated 18 and 20 weeks after completion of DNA immunization, CTL activity was found to have substantially declined; however, it was generally in the measurable range (Figure 5) and, at least at week 18, it could be substantially expanded *ex vivo *(Figure 3). Incidentally, this immunization was also followed by a reduction of FIV RNA and proviral DNA loads and by an increment of circulating CD4 T^+ ^lymphocytes, thus showing that it had exerted a beneficial effect on infection course (Pistello et al., manuscript in preparation).

## Discussion

Measurement of CTL activity is considered of paramount importance for predicting protection from infection by lentiviruses and other pathogens [1,3,4] but may be difficult due to a number of technical complications. In particular, production and use of suitable target cells generally needs expertise and facilities for using vaccinia vectors and radioactive compounds and, in non syngeneic hosts, it may be especially tricky due to the need for a constant source of MHC class 1 matched antigen-positive targets. In developing the CTL assay described here we took advantage of the method described by Kölsoy et al. [16] who, as target cells, used immortalized autologous feline primary fibroblasts infected with a recombinant vaccinia vector delivering the test antigen. Similarly infected primary feline fibroblasts have also been used as target cells but could be propagated *in vitro *for maximum 1–2 months before undergoing senescence and eventually dying off [17-19]. In their study, Kölsoy et al. demonstrated that it is possible to obtain an unlimited source of target cells retaining the same MHC class I phenotype as the cells of origin by immortalizing fibroblasts with replication competent SV40. To avoid full transformation and minimize the risk of abnormal self-antigen expression, we have instead used a molecular clone of SV40, rendered unable to replicate through an internal deletion in the large T-antigen, which immortalized primary feline fibroblasts as efficiently as wild-type SV40 (data not shown). This clone had previously been shown to transform established cell lines, such as NIH-3T3 cells, but to be unable to transform primary rat embryo fibroblasts [20-22].

Because we wished to test immortalized fibroblasts as a tool for measuring Env-specific CTL activity in FIV infected and vaccinated cats, we attempted to infect these cells with FIV, with no success. Fibroblasts from FIV infected cats also proved consistently virus-negative, thus excluding the possibility of using direct FIV infection as a means of obtaining the desired target cells. We therefore exploited a bicistronic vesicular stomatitis virus glycoprotein G (VSV-G) coated vector derived in our laboratory from FIV but carrying very little of the original virus, vector that had been previously shown to effectively introduce transgenes into primary cells of both feline and nonfeline origin [15]. The vector used carried the Env of FIV-Pet and GFP as target and reporter transgene, respectively. Immortalized fibroblasts transduced at high multiplicity with this vector were approximately 90% positive for both transgenes and proved valuable both as target cells for use in a F-CTL assay and as a stimulus for expanding CTLs *ex vivo*.

Differently from the methods that rely on vaccinia virus infection or transfection or on peptide loading used in traditional ^51^Cr assays [5,6] and in the more recently described (F-CTL) assays [7-12], this method generates target cells stably expressing the antigen of interest and the reporter molecule. This has been achieved by using a bicistronic vector that *i*) does not require the containment procedures needed for the recombinant vaccinia viruses that are frequently used to introduce transgenes in target cells, *ii*) overcomes the intrinsic resistance to transfection of primary and non-adherent cells, *iii*) delivers two large transgenes for up to 7 Kbs, *iv*) stably transduces cells, thus permitting to use the same batch of targets in follow-up studies, minimizing inter-assay variation, *v*) allows transduction of cells irrespective of animal species; *vi*) is replication incompetent, making it unnecessary ultraviolet irradiation or other treatments of the target cells to prevent infection transmission to effector cells, and last but not least, *vii*) permits the use of F-CTL assays. While most other CTL assays detect radioisotopes or cell dyes released from the target cell population in bulk and may have a high background noise due to spontaneous release of the markers, F-CTL assays rely on the actual enumeration of target cells made fluorescent by GFP, a protein that is not secreted by the cells and does not fluoresce if degraded or improperly folded in dying cells, and with the use of specific cell-specific markers may also permit phenotyping of the effector cells [6,9].

A limitation of the present study is that the F-CTL assay was not directly compared to established techniques. However, its validity was assessed by evaluating FIV Env-specific CTL activity in both acutely and chronically FIV-infected cats. The method proved valuable in either situation. During the early stages of infection, characterized by acute viral replication and vigorous cell-mediated immune responses [18,23], the assay detected levels of Env-specific CTL activity in the PBMCs of infected cats similar to those previously reported with ^51^Cr CTL assays using SV40-immortalized or primary feline fibroblasts infected by recombinant vaccinia viruses [18,24], with no need for the effector cells to be restimulated *ex vivo*, a procedure frequently necessary when using the standard Cr^51 ^assay [25]. In contrast, in the chronically infected cells we detected no measurable Env-specific CTL activity even when the effector PBMCs were restimulated *ex vivo*. This may have reflected the fact that, especially in the periphery [18], anti-FIV Env cell-mediated immune responses tend to wane with time of infection [26-28], or immunosenescence of the cats who were 10 year-old when tested [29], or a combination of these factors [30]. In any case, administration to the chronically infected cats of a DNA vaccine encoding the Env of the infecting FIV and feline granuloyte macrophage-colony stimulating factor (GM-CSF), a cytokine known to induce dendritic cell proliferation and improve antigen presentation [31], with a protocol that had elicited fairly good cell-mediated immune responses when tested in a prior prophylactic study (Pistello et al., manuscript in preparation), brought back substantial levels of CTL activity. CTL activity showed a maximum 2 weeks after completion of the vaccination schedule and then gradually decreased as previously observed for other FIV antigens [18,25], but in 2/3 animals it remained detectable for at least 20 weeks without the need for restimulating *ex vivo *their PBMC. Interestingly, this correlated with a beneficial effect of the vaccine on infection course, as demonstrated by a decrease of viral RNA in plasma and proviral load in the PBMCs and an increase of circulating CD4^+ ^T-lymphocytes (Pistello, manuscript in preparation). Of note, vaccination on an uninfected control cat elicited a modest CTL response that exceeded the cut-off only at an early post-vaccination time point only, suggesting that the vigorous CTL activity brought forth by vaccination in the chronically infected cats was mostly due to a recall of pre-existing immunity rather than to ex novo induction.

## Conclusion

The F-CTL assay described, using immortalized feline fibroblasts stably transduced with the test antigen and the GFP reporter as target cells, has proven valuable in evaluating Env-specific CTL activity in FIV infected and vaccinated cats. Given the versatility of the bicistronic vector used for producing target cells, the method has the potential to be used for a wide array of antigens and hosts.

## Methods

### Plasmids and vectors

The GFP encoding plasmid pcDNA3-GFP was previously described [25]. Plasmid pACTSV2 encoding a replication defective SV40 genome with a functional small T and a large T antigen mutated by an internal deletion of 43 amino acids [20] was a kind gift of Dr. Mauro Tognon (University of Ferrara, Italy). Construction of the bicistronic vEnv-GFP and monocystronic vGFP vectors, derived from molecular clone p34TF10 of FIV-Pet, and of the packaging (pΔenv1) and VSV G-protein (pVSV-G) plasmids has been described [15]. Briefly, vEnv-GFP delivers the target Env of FIV-Pet and the reporter GFP placed under the control of the FIV LTR and the cytomegalovirus promoter, respectively (Figure 1). vGFP has the same architecture except that it encodes the reporter alone. Pseudotyped vector particles were generated in human epithelial 293T cells that were co-transfected with pΔenv, pVSV-G and either vEnv-GFP or vGFP, exactly as described [15]. Two days later, approximately 80% of the cells were GFP positive by FACS analysis, regardless of whether exposed to vEnv-GFP or vGFP. The pseudotyped virus preparations used for transduction consisted of 293T cell culture supernatants harvested on day 2, filtered through a 0.45 μm filter (Nalge Europe, Neerijse, Belgium), and titered for TU in 293T cells as described [15]. They were either used immediately or stored in aliquots at -80°C until use, with no appreciable differences in transduction efficiency.

### Animals

The SPF cats (IFFA Credo, Lyon, France) used were housed individually in our climatized animal facility in accordance with European Community guidelines, had *ad libitum *access to fresh water and a proprietary brand of cat food, and were sedated with ketamine/diazepam intramuscularly prior to any procedure. Cats GN, GO, GP and GQ were FIV infected after entering the study (acutely infected cats). Infection was performed intraperitoneally when 18-month old with 1 ml of a stock of pooled plasma from FIV-Pet infected cats diluted to contain 10 cat infectious doses 50% per ml. Three further cats (BR, BS and CF) were already infected when the study started (chronically infected cats). These had been infected exactly as above 5 years earlier and were 10-year old at the start of the study. An additional 10-year old cat (BO) that had been similarly inoculated but had escaped infection by all parameters served as uninfected control for the latter animals. Because the chronically infected cats showed no detectable Env-specific CTL activity (see below), they were boosted by DNA immunization with p-Env-GMCSF, a plasmid expressing the Env of FIV-Pet and feline GM-CSF, as an immunoadjuvant (Pistello et al., manuscript in preparation). This was inoculated intramuscularly at doses of 300 μg (300 μl) into both hind legs (150 μl/site) 20 minutes after rubbing the shaved skin with Aldara (Graceway Pharmaceuticals, Bristol, TN, USA), a topical Imiquimod containing cream known to exert an adjuvant effect. Infection of cats was monitored as described [25] by testing for presence and loads of FIV RNA in plasma and proviral DNA in the PBMCs, anti-FIV antibody in serum and counting peripheral CD4^+ ^and CD8^+ ^T-lymphocyte.

### Preparation of target cells

Primary fibroblasts were obtained from skin biopsies of the study cats with sterile, 4 mm diameter, disposable skin biopsy punches (Steifel Laboratories, Segrate, Italy) and cultured in 6-well plates with minimum essential medium-α (Sigma-Aldrich, Milan, Italy) supplemented with ribonucleosides and deoxyribonucleosides, 10% fetal bovine serum (FBS; Sigma-Aldrich), 200 U/ml penicillin and streptomycin (Eurobio, Labtek, Milan, Italy) and 2 mM L-glutamine (Sigma-Aldrich). Around day 20, when the cultures were confluent, the cells were immortalized by transfecting 4–5 × 10^4 ^cells, seeded the day before in 10 cm Petri dishes, with 2 μg pACTSV2 [20,21] and using a calcium phosphate method previously described [15]. This led to the appearance of typical signs of cell immortalization (i.e. unlimited replication, loss of inhibition contact and growth in low FBS medium) in approximately one week and to generalized immortalization in 10 to 15 days. The fibroblasts thus immortalized were then seeded into 6-well plates at 3 × 10^5 ^cells/well and, 18 hours later, transduced with 3 × 10^6 ^TU of vEnv-GFP (Env^+^GFP^+ ^fibroblasts) or, as antigen control, with a similar dose of vGFP (GFP^+ ^fibroblasts). Expression of the transgenes was monitored 2 days post-transduction and then twice weekly for up to 4 weeks by flow cytometry (GFP) and Western blot (Env). Finally, the target cells were expanded and frozen for further use.

### Analysis of the target cells for Env expression

Env expression by the target cells was evaluated by Western blot analysis. Briefly, 1 × 10^5 ^cells were lysed in 100 μl lysis buffer (10 mM Tris-HCl pH 7.5, 150 mM NaCl, 2 mM EDTA, 0.5% Nonidet P-40), mixed with 2× loading buffer, and loaded (20 μl) on a 10% polyacrylamide gel (Bio-Rad Laboratories, Milan, Italy). Proteins were transferred onto a nytran C-extra nitrocellulose membrane (General Electric Healthcare, Milan, Italy) and reacted with the anti-FIV surface glycoprotein monoclonal antibody vpg71.2, kind gift of Dr. Brian Willett, University of Glasgow, Glasgow, UK. Detection was done with a rabbit anti-mouse IgG horseradish peroxidase-conjugated monoclonal antibody (Sigma-Aldrich). The FIV chronically infected feline lymphoid cell line FL-4 [32] was used as positive control.

### Effector cells

Effector cells were PBMCs separated from 20 ml of EDTA or citrate anticoagulated blood of the study cats on Ficoll/Hystopaque 1077 (Sigma-Aldrich), washed in PBS, viability counted with trypan blue, and suspended in RPMI 1640 (Sigma Aldrich) supplemented with 2 mM L-glutamine, 200 U/ml penicillin and streptomycin and 10% FBS. PBMCs were used as such or after *ex vivo *restimulation. For restimulation, 5 × 10^6 ^PBMCs were cultured in RPMI 1640 supplemented as above in 6-well plates that had been seeded with 1 × 10^5 ^Env^+^GFP^+ ^or, as a negative control, GFP^+ ^fibroblasts 1 day earlier. After partial substitution of the culture medium with fresh medium containing 20 U/ml human interleukin-2 (Roche Diagnostics, Milan Italy) at day 3, the restimulated cells were harvested on day 5, viability counted, and suspended as above. In one experiment, restimulation was also carried out by incubating the PBMCs with immortalized fibroblasts that had been loaded for 1 hour with 10 μM (final concentration) pooled 21-mer peptides overlapping by 11 amino acids and encompassing the entire sequence of the FIV-Pet Env. The peptides had been synthesized by Espikem (Florence, Italy) with *fmoc *chemistry and were >95% pure.

### F-CTL assay

The day before the assay, 1 × 10^4 ^Env^+^GFP^+ ^fibroblasts and, as negative control, GFP^+ ^fibroblasts derived from at least two distinct cats were individually seeded onto a 48-well plate and incubated overnight to allow complete adherence. PBMCs, either freshly separated or restimulated *in vitro*, were added at E:T ratios of 10:1 and 50:1 onto autologous and heterologous fibroblasts. After overnight incubation at 37°C in a humidified CO_2 _atmosphere, the PBMCs were removed, and the adherent cells extensively washed, trypsinized and fixed for FACS analysis. Analysis was performed using a FACScan cytometer (Becton Dickinson). The forward scatter (FSC) threshold was set to measure nonviable cells as well and to acquire 1 × 10^6 ^events/sample. Cellular debris were then left out by gating in FSC-SSC dot plots during data acquisition. Numbers of viable fluorescent cells were plotted in a number of events/fluorescence intensity (FL-1) histogram, and acquired data were analyzed using CellQuest Pro software (version 3.4). Samples were analyzed in triplicate and percent specific anti-Env CTL activity was calculated using the formula: (% fluorescent target cells incubated alone - % fluorescent target cells incubated with autologous effector cells) - (% fluorescent target cells incubated alone - % fluorescent target cells incubated with heterologous effector cells)/% fluorescent target cells incubated alone × 100. In preliminary studies aimed at evaluating nonspecific target cell lysis, PBMCs were also incubated with autologous and heterologous GFP^+ ^positive cells. Nonspecific lysis (background), calculated using cat BR, BS, CF, and BO cells in three independent experiments, averaged 2.9%, and standard deviation (SD) was 1.0. Thus, values of lysis above 6% (i.e. background + 3 × SD) were considered positive.

## Authors' contributions

FB and EZ devised the experiments, produced the vector constructs and performed *in vitro *and *in vivo *tests. FC established the primary cell lines and together with EZ immortalized and transduced the cells. AM vaccinated and monitored clinical conditions and hematological parameters of animals. GF helped to the set up the F-CTL assay and carried out flow cytometry analyses together with EZ and FC. MB and MP wrote the manuscript. All authors read and approved the final manuscript.
